# Revolutionizing Surgical Care: The Power of Enhanced Recovery After Surgery (ERAS)

**DOI:** 10.7759/cureus.48795

**Published:** 2023-11-14

**Authors:** Reda H Mithany, Nesma Daniel, M Hasaan Shahid, Samana Aslam, Mark Abdelmaseeh, Farid Gerges, Muhammad Umar Gill, Shenouda B Abdallah, Abdul Hannan, Muhammad Talha Saeed, Mina Manasseh, Mohamed S Mohamed

**Affiliations:** 1 Department of General and Emergency Surgery, Kingston Hospital National Health Service (NHS) Foundation Trust, Kingston Upon Thames, GBR; 2 Medical Laboratory Science, Ain Shams University, Cairo, EGY; 3 Surgery, Glangwili General Hospital, Carmarthen, GBR; 4 General Surgery, Lahore General Hospital, Lahore, PAK; 5 General Surgery, Faculty of Medicine, Assuit University, Assuit, EGY; 6 Department of General and Emergency Surgery, Kingston Hospital National Health Service (NHS) Foundation Trust, London, GBR; 7 Accident and Emergency, Kings College Hospital National Health Service (NHS) Foundation Trust, London, GBR; 8 Surgery, Jaber Al-Ahmad Hospital, Kuwait, KWT; 9 Internal Medicine, Prince Philip Hospital, Llanelli, GBR; 10 General Surgery, Torbay and South Devon National Health Service (NHS) Foundation Trust, Torquay, GBR; 11 Orthopaedics, King’s College, London, GBR

**Keywords:** eras protocol, patient-centered approach, multimodal pain management, enhanced recovery after surgery (eras), perioperative care

## Abstract

The development of Enhanced Recovery After Surgery (ERAS) has brought about substantial transformations in perioperative care, substituting conventional methods with a patient-centric, evidence-based strategy. ERAS protocol adopts a holistic approach to patient care, which includes all stages preceding, during, and following the operation. These programs prioritize patient-specific therapies that are tailored to their specific requirements. Nutritional assessment and enhancement, patient education, minimally invasive procedures, and multimodal pain management are all fundamental components of ERAS. ERAS provides a multitude of advantages, including diminished postoperative complications, abbreviated hospital stays, heightened patient satisfaction, and healthcare cost reductions. This article examines the foundational tenets of ERAS, their incorporation into the field of general surgery, their suitability for diverse surgical specialties, the obstacles faced during implementation, and possible directions for further investigation, such as the integration of digital health technologies, personalized patient care, and the long-term viability of ERAS protocols.

## Introduction and background

The implementation of Enhanced Recovery after Surgery (ERAS) has significantly transformed perioperative care by introducing a patient-centric, evidence-based approach that has previously been unheard of. Historically, standard surgical procedures entailed extended bed rest, protracted fasting, and bowel preparation preceding the procedure. These measures resulted in complications, prolonged hospital stays, and increased healthcare expenditures. Since the late 1990s, ERAS protocols have become increasingly well-known, placing significant emphasis on the cooperative endeavors of a multidisciplinary group including dietitians, surgeons, anesthesiologists, and nurses. The principal aim of this collaborative endeavor is to enhance the quality of surgical care as a whole, including the preoperative, intraoperative, and postoperative stages, by emphasizing individualized treatment plans that address the specific requirements of each patient [1].

The fundamental principle underlying Enhanced Recovery after Surgery (ERAS) protocol is the critical requirement to improve surgical outcomes and ensure patient adherence via a holistic strategy for perioperative care. ERAS prioritizes the methodical assessment and surveillance of nutritional and functional status, recognizing the potential hazards that may arise from disregarding conditions such as sarcopenic obesity, which can lead to complications after surgery. ERAS is in accordance with metabolic and nutritional standards and seeks to integrate nutrition into a comprehensive strategy for patient care by decreasing preoperative fasting, encouraging prompt oral feeding resumption, and delivering expeditious nutritional intervention in cases where nutritional hazards are detected. The aforementioned strategy is of paramount importance in reducing postoperative complications and enhancing patient compliance, particularly among gastrointestinal cancer patients undergoing neoadjuvant therapy and where progressive nutritional deficiencies may become more severe. Novel approaches such as "prehabilitation" are utilized to prime patients for ERAS, while the incorporation of oral nutritional supplements, metabolic conditioning, and immunonutrition serves to enhance the overall continuum of perioperative care. This emphasizes the significance of an integrated, evidence-based approach to recovery and surgery [2]. The objective of this article is to provide a thorough examination of Enhanced Recovery After Surgery (ERAS), encompassing its underlying principles, practical application, results, obstacles, and potential. The ultimate objective of this initiative is to educate researchers and healthcare professionals about the revolutionary capabilities of ERAS in enhancing patient outcomes and the surgical experience, thereby motivating additional progress in the field of surgical care.

## Review

Definition and principles of ERAS

Enhanced Recovery After Surgery (ERAS) is a systematic, evidence-based, multimodal approach to perioperative care in the healthcare industry that aims to maximize the recovery of patients undergoing surgery. ERAS program comprise meticulously customized interventions and protocols that address particular surgical procedures throughout the preoperative, intraoperative, and postoperative stages [3]. ERAS is a perioperative care approach that prioritizes the needs and desires of the patient by streamlining the entire surgical procedure in order to accelerate postoperative recovery and enhance patient outcomes. A multidisciplinary approach is incorporated into the ERAS core principles, which includes patient education to equip individuals with information regarding the surgical procedure and their responsibility in the recovery process. Preoperative health optimization, minimally invasive techniques, personalized anesthesia, preventive pain management, early initiation of oral nutrition, prompt mobilization, careful fluid management, reduced reliance on drains and tubes, standardized postoperative care protocols, and opioid-sparing pain management strategies are all encompassed within these principles. They require the collaboration of healthcare professionals from diverse disciplines. Although individual elements vary according to hospital policies and geographical location, these fundamental principles form the basis of ERAS and have been shown to be efficacious in diminishing patient distress, hospital stays, and complications, all while enhancing overall surgical results [4].

ERAS originated in 1997 when a group of visionary general surgeons, primarily from Northern Europe and led by Henrik Kehlet, pioneered the concept during their experiences with colorectal fast-track surgery. Their collaborative research sought to enhance perioperative care for patients undergoing open colorectal procedures by mitigating surgical stress, optimizing nutritional status, utilizing non-opioid analgesia, and advocating early postoperative nutritional intake. As their initiative expanded to include professionals from various surgical subspecialties and regions, significant improvements in postoperative recovery became evident. In 2010, the ERAS Society was formally established as an international non-profit medical academic society, furthering its mission of disseminating guidelines, organizing an annual international congress, and launching the ERAS Implementation Program (EIP). The EIP, initially launched in Sweden and later embraced globally, has played a pivotal role in implementing ERAS across diverse healthcare settings, with the ERAS Interactive Audit System (EIAS) ensuring real-time quality control. ERAS continues to evolve, representing a flexible approach to patient care that has fostered a paradigm shift in surgical practice and research, transcending geographic boundaries [5].

Implementation of ERAS in general surgery

The practical implementation of ERAS in general surgery is contingent upon a robust multidisciplinary approach that underscores seamless collaboration among diverse healthcare professionals. This holistic strategy entails harmoniously engaging surgeons, anesthesiologists, nurses, dietitians, physiotherapists, and allied health experts to optimize patient care throughout the perioperative journey. Each team member assumes a pivotal role in this continuum, commencing with the preoperative phase, wherein nutrition assessment and prehabilitation strategies are meticulously employed. The intraoperative phase employs minimally invasive techniques and custom-tailored analgesia regimens to attenuate surgical stress. The postoperative phase, equally pivotal, emphasizes early mobilization, nutrition support, and opioid-sparing pain management. The collaborative effort described in this study aims to provide comprehensive and evidence-based care in general surgery. This approach has reduced hospital stays, lowered the incidence of complications, and improved overall outcomes [6].

ERAS in general surgery (for both elective and emergency surgeries) is a comprehensive approach that entails thorough preoperative preparation and patient education. During this crucial stage, a thorough evaluation of the patient's overall health and nutritional condition is conducted to detect any potential risk factors and develop a personalized care plan. Patient education is vital during this stage since it empowers patients to understand comprehensively. Patients are provided with information regarding the advantages of early mobilization, the importance of maintaining enough nutrition, and the prudent utilization of opioids for the management of pain. The process of preoperative counseling has been found to exert several positive effects on patients, including increased empowerment, reduced anxiety, and improved adherence to ERAS protocols. These factors eventually contribute to better postoperative recovery and enhanced surgical outcomes [7].

The ERAS program places significant importance on reducing the physiological stress response associated with surgical procedures and facilitating a faster recovery process. In the operating room, this translates into strategies such as maintaining normothermia, judicious fluid management, and optimized pain management. Techniques such as epidural anesthesia, regional nerve blocks, or intravenous lidocaine infusions are employed to reduce the reliance on opioids, which are known to retard recovery. Furthermore, maintaining net-zero fluid balance and avoiding excessive intravenous fluids contribute to superior postoperative outcomes. In essence, the intraoperative phase of ERAS revolves around attenuating surgical stress and facilitating a seamless transition to the postoperative period, aligning with the overarching objective of enhancing patient recovery in general surgery [8].

**Figure 1:**
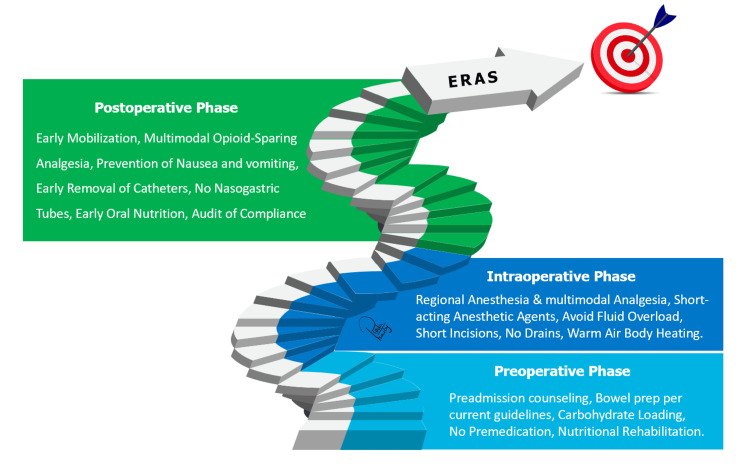
Enhanced Recovery After Surgery (ERAS) Protocol Figure created by author Reda Harby

Postoperative care pathways are meticulously structured to expedite recovery and ensure a smooth transition from the operating room to the postoperative phase. Key elements of these pathways encompass early mobilization, vigilant pain management focusing on non-opioid analgesics, and the prompt resumption of oral nutrition. Patients are encouraged to initiate mobility and return to their usual activities as soon as possible to avert complications such as deep vein thrombosis and muscle atrophy. Moreover, avoiding protracted fasting and the early reintroduction of a regular diet significantly reduce the stress response to surgery and facilitate recovery (Figure 1). Vigilant monitoring and timely intervention in the face of potential complications are essential facets of these pathways. ERAS-driven postoperative care pathways are meticulously designed to curtail hospital stays and hasten patients' return to their everyday routine [9].

Outcomes and benefits of ERAS

These comprehensive, evidence-based protocols have significantly reduced postoperative complications, shorter hospital stays, and expedited recovery. By optimizing perioperative care, ERAS has reduced surgical stress, leading to decreased complications such as infections and wound-related issues. This not only reduces the benefit to patient health but also significantly reduces healthcare expenditures. The hallmark of ERAS is shorter hospital stays, allowing patients to return quicker to their everyday lives and alleviating the strain on healthcare facilities [10,11].

Moreover, ERAS has yielded enhanced patient satisfaction and quality of life. With diminished postoperative pain and a faster recovery trajectory, patients experience reduced discomfort and tend to report positive experiences with their surgical procedures. These outcomes and the potential for an earlier resumption of work and daily activities have contributed to an elevated sense of well-being and satisfaction among patients. In summary, ERAS programs in general surgery have consistently demonstrated improved patient outcomes, shorter hospital stays, reduced complications, and heightened patient satisfaction, thereby enriching both clinical care and the cost-effectiveness of the healthcare system [3,10].

ERAS in different surgical specialties

The ERAS protocol encompasses several pivotal components spanning various stages of the surgical process. In the preoperative phase, patients receive counseling, abstain from smoking and alcohol use, address anemia, discontinue hormone therapy and oral contraceptives, partake in nutritional counseling, and avoid bowel preparation. Intraoperatively, antibiotics are administered, focusing on maintaining normothermia and proper fluid balance. In the postoperative phase, extended venous thromboembolism prophylaxis, effective pain management, avoidance of drains, maintenance of proper fluid balance, early mobilization, removal of urinary catheters, and the early initiation of a high-protein diet are implemented to enhance recovery and minimize complications [12].

ERAS protocols transcend specific surgical domains and have been adeptly incorporated into diverse surgical specialties to enhance patient outcomes and expedite postoperative recovery. The ensuing discussion offers a concise overview of the application of ERAS principles across various surgical realms.

In orthopedic surgery, encompassing procedures such as joint replacements and spinal surgeries, ERAS protocols are meticulously tailored to address postoperative pain, curtail hospitalization durations, and expedite patient mobilization and resumption of regular activities. These protocols prioritize pain management, complication reduction, and facilitating a swifter recuperation trajectory. This is achieved by judicious utilization of minimally invasive surgical techniques, multimodal pain management strategies, and the early institution of rehabilitative measures, collectively fostering an enhanced patient recovery experience within orthopedic surgery [13].

Gynecological surgery, which includes procedures such as hysterectomies and pelvic surgeries, employs ERAS protocols thoughtfully constructed to mitigate postoperative pain, reduce hospital stays, and expedite recovery. Central components of these protocols typically involve the use of minimally invasive surgical approaches, effective pain management modalities, prompt, patient ambulation, and a dedicated emphasis on preserving and restoring pelvic functionality, all contributing to the attenuation of postoperative complications [14].

Urological surgeries, encompassing procedures like prostatectomies, cystectomies, and renal interventions, adhere to ERAS principles aimed at minimizing surgical trauma, reducing complication rates, and expediting patient recovery. These protocols encompass the judicious utilization of minimally invasive surgical techniques, the early removal of urinary catheters, tailored pain management strategies, and the prompt restoration of normal urinary function. These facets collectively enhance the surgical experience in urological interventions [15].

In cardiothoracic surgery, including procedures such as coronary artery bypass grafting (CABG) and heart valve surgeries, ERAS protocols undergo tailored adaptation to enhance surgical outcomes. These adaptations encompass strategies designed to mitigate surgical stress, optimize cardiopulmonary function, and diminish the incidence of postoperative complications. Key elements of these protocols include the judicious use of minimally invasive surgical techniques, precise fluid management, early extubation, and the prompt mobilization of patients. These measures collectively contribute to an enhanced postoperative recovery trajectory in the context of cardiothoracic surgery [16].

Challenges and barriers to ERAS implementation

Implementing ERAS protocols poses several obstacles and constraints contingent upon elements such as healthcare environments, patient demographics, and the characteristics of the surgical procedures. One of the primary challenges in implementing Enhanced Recovery After Surgery (ERAS) protocols is the presence of resistance to change. This resistance is frequently observed among healthcare workers who are accustomed to standard care practices and are hesitant to embrace the foreign principles associated with ERAS. Insufficient awareness and education pertaining to ERAS can hinder the successful application of this approach, emphasizing the necessity for educational programs to bridge the existing knowledge gap. Challenges pertaining to patients encompass various aspects, including non-adherence to preoperative instructions, unrealistic patient expectations, and reluctance to modify behaviors that impact surgery outcomes. To effectively tackle these difficulties, it is imperative to prioritize patient education and participation. The presence of robust data collecting and monitoring systems is crucial in assessing the effectiveness of ERAS implementation. However, the lack of such systems might impede the measurement of outcomes and restrict the ability to make informed adjustments. Resistance to standardization, often from the perception that ERAS protocols may be overly prescriptive, poses a concern among clinicians. This perception may be due to the variability in different surgical procedures, necessitating adaptable ERAS protocols, and inadequate training can hinder healthcare professionals from effectively implementing ERAS. Additionally, cultural beliefs and societal factors may influence patient adherence to ERAS recommendations, including dietary changes and smoking cessation [17-19].

Comparative analysis of ERAS protocols

**Figure 2:**
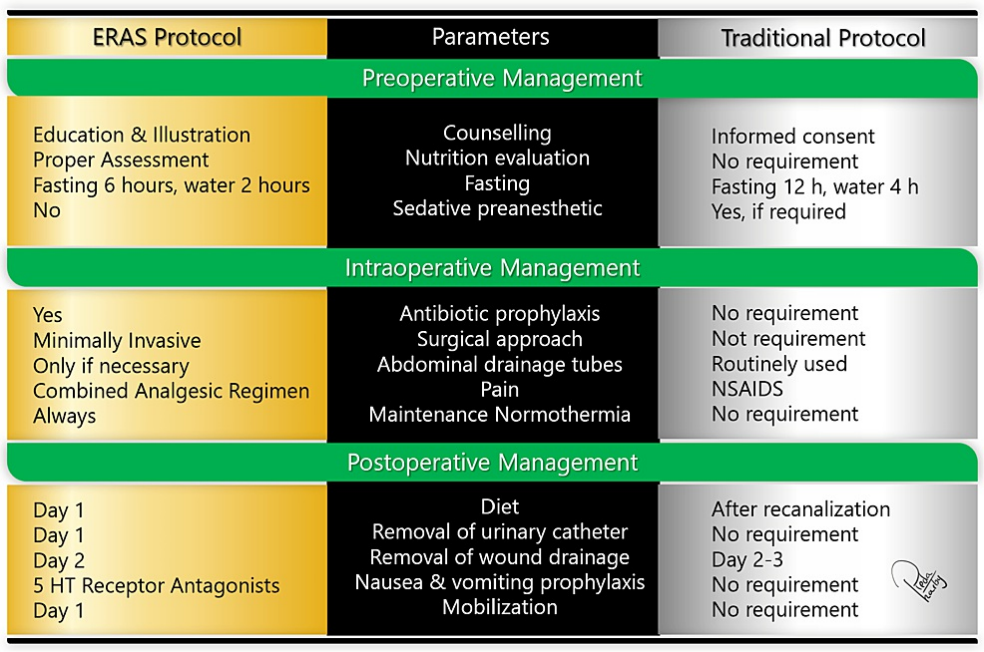
Enhanced Recovery After Surgery (ERAS) versus traditional postoperative care Figure created by author Reda Harby.

A comparative analysis of ERAS protocols in contrast to traditional care reveals that ERAS is more effective than traditional care in improving postoperative recovery. ERAS protocols, characterized by their evidence-based and multimodal approach, are meticulously designed to optimize patient outcomes and expedite post-surgical recovery. They prioritize preoperative patient education, minimal fasting periods, early mobilization, and customized pain management strategies. In contrast, traditional care often adheres to established practices involving prolonged fasting, stringent fluid management, and the routine use of surgical drains and nasogastric tubes. Pain management in traditional care predominantly relies on opioid-based interventions. The comparative analysis underscores the advantages of ERAS protocols, notably their demonstrated ability to reduce hospital stay complications and enhance overall patient satisfaction. ERAS primarily focuses on mitigating the surgical stress experienced by patients and expediting their return to normal activities. In contrast, traditional care may inadvertently prolong patient recovery times and contribute to heightened discomfort (Figure 2) [20,21].

Furthermore, variations within ERAS protocols are essential to accommodate the diverse demands of different surgical specialties and procedures. While the fundamental principles, including early mobilization, optimized nutrition, and individualized pain management, remain consistent, specific elements are tailored to suit each surgical context's unique requirements. This customization is especially pronounced in orthopedics, gynecology, urology, and cardiothoracic surgery. For example, urological surgery emphasizes early catheter removal, while orthopedic procedures prioritize rapid ambulation and specialized pain control, particularly in joint replacement surgeries [22,23].

Additionally, patient selection and individualization are pivotal aspects of ERAS. These protocols acknowledge that not all patients are ideal candidates and that a personalized approach is essential. Criteria for patient selection may encompass factors such as age, comorbidities, and psychosocial support, optimizing care based on each patient's unique needs. On the other hand, conventional healthcare may employ a standardized approach, disregarding the unique variances in patients' health conditions and social contexts. As a result, ERAS demonstrates a dedication to patient selection and individualization, which contributes to the provision of customized treatment. This approach eventually improves patient outcomes and enhances surgical experience [24].

Future directions and research gaps

In the dynamic realm of ERAS, numerous nascent patterns are poised to influence the trajectory of surgical care in the foreseeable future. It is essential to highlight that patient-centered treatment is anticipated to be prioritized, specifically catering to each individual's unique preferences, values, and objectives. The implementation of personalization inside the ERAS framework has the potential to enhance patient satisfaction and engagement. Moreover, the adoption of digital health solutions, telemedicine, and wearable gadgets for remote monitoring and follow-up is becoming increasingly prominent. Real-time data gathering and communication can enhance the overall postoperative recovery experience. The concept of prehabilitation, which involves preparing patients physically and mentally before surgery, is likely to become a more integral part of ERAS, allowing patients to optimize their health and resilience for improved outcomes. Tailoring nutritional interventions based on a patient's genetic makeup, known as nutrigenomics, presents a promising avenue for enhancing postoperative recovery. Understanding how genetic predispositions influence nutritional needs can be a future direction. Furthermore, sustainability is emerging as a relevant consideration in ERAS, emphasizing adopting environmentally friendly practices, such as reducing single-use plastics and optimizing resource utilization, in alignment with broader sustainability goals [25,26].

In the realm of ERAS, several areas demand further research to advance our understanding and refine the implementation of these protocols. One such area pertains to the assessment of long-term outcomes. While numerous studies have focused on short-term results, investigating how ERAS impacts patients' quality of life, long-term complications, and disease-free survival in cancer patients is critical for a comprehensive understanding. Evaluating the cost-effectiveness of ERAS programs is another crucial avenue for research. This should encompass immediate healthcare costs, broader economic implications, and societal benefits. Research into the most effective strategies for implementing ERAS across diverse healthcare settings and surgical specialties is needed to overcome barriers and devise practical solutions. Additionally, optimizing pain management within ERAS, including exploring non-pharmacological interventions, is vital to reduce reliance on opioids and enhance patient comfort. A focus on the patient experience within ERAS offers valuable insights into tailoring care to individual needs and preferences, thus enhancing overall satisfaction and compliance.

## Conclusions

ERAS represents a transformative paradigm shift in perioperative care, offering a holistic and evidence-based approach to enhance patient outcomes and expedite postoperative recovery. By focusing on individualized care, multidisciplinary collaboration, and innovative strategies, ERAS has consistently shown its capacity to reduce complications, hospital stays, and healthcare costs while improving patient satisfaction and quality of life. Future research must explore long-term outcomes, cost-effectiveness, implementation strategies, pain management optimization, and patient experience to overcome challenges and refine ERAS implementation. As ERAS continues to evolve, patient-centered care, digital health integration, and sustainability practices are expected to shape the future of surgical care, ensuring that ERAS remains at the forefront of perioperative medicine.
